# Supplementary material for: Perceived overqualification as a double-edged sword for employee creativity: The mediating role of job crafting and work withdrawal behavior
Source: PLoS One. 2024 Jun 17;19(6):e0304529. doi: 10.1371/journal.pone.0304529 (PMC11182559; doi:10.1371/journal.pone.0304529)
Supplement: Attachment — Submitted filename: Response to Reviewers.docx [file pone.0304529.s002.docx]

Dear Editors and reviewers,

Thank you for your valuable feedback and comments on my manuscript submission.

I deeply understand the existing shortcomings and the necessity to address them comprehensively. I will critically revisit the manuscript and make the required improvements based on the reviewers' suggestions to meet the standards for publication in your esteemed journal.

Additionally, I will include a detailed response outlining how each point of feedback has been addressed and incorporated into the revised submission.

**The detailed changes we made are as follows:**

**Abstract: The abstract must contain the main purpose of the paper, the research method used in the research and the main contributions.**

**Response:**

Thank for your comment. Based on your suggestion, we have reorganized the abstract section. The main purpose of the paper is "In this study, we attempt to clarify the relationship between perceived overqualification and employee creativity from an integrated perspective, exploring both positive and negative pathways. " The research method used in the research is "Based on 487 valid samples collected in three stages, empirical analysis is conducted". And the main contributions contains "This study provides a theoretical explanation for the inconsistent results of previous studies on the relationship between perceived overqualification and employee creativity from a resources perspective; The study conclusion deepens the research on the mechanism of the influence of the perceived overqualification on employees' work behavior, and provides practical enlightenment for the organization and management of employees with excess qualification. "

**Introduction: It would be very useful to add the "Introduction" section and the purpose, objectives and hypothesis of the** **research. I consider that a weak point of the paper is that the authors did not show** **the novelty of the paper compared to other works. That is why, I consider that the introduction should specify the novelty of the paper compared to other papers published in this area.**

**Response:**

Thanks for your comment. Based on your suggestion, we have reorganized the introduction section. We tired our best to create tension. In the first paragraph of the INTRODUCTION in the new manuscript, we have reviewed the current research of perceived overqualification and presented research gap. In the second paragraph of the INTRODUCTION in the new manuscript, we explain the reason for looking at the impact of perceived overqualification on creativity. In the third paragraph of the INTRODUCTION in the new manuscript, we explain the reason for choosing job crafting and work withdrawal behavior as mediating variables. And then, in the fourth paragraph of the INTRODUCTION part in the new version of the manuscript, we added the explanation of choose organizational identification as a moderating variable. We introduced the new set of mediators, the outcome and the moderator in INTRODUCTION.

**Theoretical Background and Hypotheses: The literature support for your first three hypotheses is not sufficient; similar for H6.**

**Response:**

Thanks for your comment. Based on your suggestion, we supplemented H1-H3 and H6 to make up for the lack of references, such as: "Individuals with more resources have more opportunities to invest in resources, forming a resource gain spiral. Therefore，overqualified employees have more opportunities to use their surplus skills to proactively reinvent their jobs to create a better work environment (Wu et al., 2015), thus reducing the difference between reality and their ideal job."

**Method: In regard to your methodology, it should include the following information: (1)** **participants and procedure; (2) the** **measures that you used; (3) the** **analysis strategy; (4) the results where you need to enclose descriptive statistics and hypotheses testing. Please add the missing information.**

**Response:**

Thanks for your comment. Based on your suggestion, we have reorganized the method section.

(1) The participants and procedure of the paper is "The research was conducted in five companies in eastern China, and the process was as follows: First, after seeking permission from company leaders, the researchers contacted the department heads to introduce the purpose of the study and explain the potential benefits of the study……" (2) The measures of the paper are "All of the measurement scales were established well and drawn from the literature. All items used the same seven-point Likert scale format ranging from 1 (strongly disagree) to 7 (strongly agree).……"

(3) The analysis strategy of the paper is "Firstly, this study conducted confirmatory factor analysis and common method bias test using Amos 22.0.…… "

(4) The results of the paper please see the results section, including CFA、CMB、Descriptive Statistical Analysis and Hypothesis Testing.

**Result:**

**1. Authors must specify the software used (STATA, Eviews, SPSS, etc.).**

**Response:**

Thanks for your comment. In the METHOD part, we have added the Analytical Strategy. In this section, we have provided a detailed explanation of the software we were using (Amos22.0, SPSS22.0, Mplus8.3).

**2. It is very important for the authors to analyze the descriptive analysis (with Kurtosis test, Jarque Berra test and interpretation, Skewness and Kurtosis interpretation). In the same time the correlation analysis and the VIF test are very important in this research.**

**Response:**

Thanks for your comment. Based on your suggestion, we have added the descriptive analysis in the method section. "In addition, as shown in Table 3, according to the skewness values, each variable is less than 0, and the distribution is negative skewness, with the tail on the left and the peak tip on the right. In terms of the kurtosis values, since all the variables were lower the threshold of 3……"

**3. What are the results of your Pilot study?**

**Response:**

Thank you for your question. In this study, we did not conduct a pilot study. We agree that it is effective to adopt a pilot study. In future studies, we will conduct a pilot study before the formal study.

**4. As for your Results chapter, please add literature accepted thresholds for all the measures used.**

**Response:**

Thanks for your comment. In response to your feedback, we have made the following revisions in the results section:

Marked acceptable thresholds for each indicator in CFA, such as: "If RMSEA is less than 0.06 and IFI, TLI, and CFI are greater than 0.9, it indicates that the model has a high degree of fit (Hair)."

**Conclusion and Discussion:**

**1. Please add a** **summary in regard to your Hypotheses results and see to it in the light of previous research.**

**Response:**

Thanks for your comment. We have added the summary in regard to our Hypotheses results, please see "The results show that: First, the "double-edged sword" effect of perceived overqualification has been verified. The impact of the sense of overqualification on creativity includes two ways……"

**2. I suggest to the authors that the last section Conclusions and Policy Implications.**

**Response:**

Thanks for your comment. We have adjusted the last section to Conclusions and Policy Implications.

**References: I recommend the authors to refer to other recent works indexed in Web of Science, because only some cited works is not enough for a scientific paper. In my opinion, the authors must cite other** **papers regarding this subject and other subjects such as: tax compliance, economic growth etc.**

**Response:**

Thanks for your comment. We have added a number of articles in this and other subjects, including those you recommended.

Thanks again for the valuable opinions of the experts on this article; I wish you good health and success in your work!

Sincerely,

All authors
